# The Use of Combined Oral Contraceptives for ˃6 Months Is Not Associated with Body Fat or Bone Density, Regardless of Dietary Differences—A Pilot Study

**DOI:** 10.3390/medicina62010127

**Published:** 2026-01-08

**Authors:** Anna-Liisa Tamm, Ülle Parm, Jelena Sokk, Siret Läänelaid, Aivar Orav, Kaido Liiv, Ester Jaansoo, Marit Salus, Ivi Vaher, Kevin Köster, Robin-Voldemar Rõžko, Mildred Mustkivi, Taimi Taimalu, Kristiina Virro

**Affiliations:** 1 Physiotherapy and Environmental Health Department, Tartu Applied Health Sciences University (TAHSU), 50411 Tartu, Estonia; 2 Nursing and Midwifery Department, Tartu Applied Health Sciences University (TAHSU), 50411 Tartu, Estonia; 3 Radiography and Biomedical Laboratory Science Department, Tartu Applied Health Sciences University (TAHSU), 50411 Tartu, Estonia; 4 Vocational Education Department, Tartu Applied Health Sciences University (TAHSU), 50411 Tartu, Estonia

**Keywords:** lifestyle, nutrition, oral contraceptives, body composition, bone health

## Abstract

*Background and Objectives*: There is a lack of information on the dietary intake of long-term combined oral contraceptive users (COC-users) in relation to their body composition. The aim of the pilot study was to determine the food consumption of young women using COCs for >6 months and its relationship to body composition compared to non-users. *Materials and Methods*: A total of 45 healthy women (21.8 ± 1.7 years) were divided into COC non-users (*n* = 19) and users of a low (≤20 μg) ethinylestradiol (EE) dose (*n* = 18) and a medium (30–35 μg) EE dose (*n* = 8). Anthropometric data, body composition, physical activity levels, three-consecutive-days of dietary records, and serum concentrations of calcium, magnesium, and vitamin D were assessed. Statistical methods included chi-squared, Fisher’s exact test, *t*-test, Mann–Whitney U test, Spearman’s correlation, and multiple linear regression. *Results*: There were no differences between COC-users and non-users in terms of physical activity, intake of micronutrients, body mass index, body fat percentage (BF%), or bone mineral density (BMD). COC-users consumed a higher percentage of energy from fats (*p* = 0.02) and had higher serum vitamin D levels (*p* < 0.01). BF% was negatively associated with BMD (coefficient −0.008; *p* = 0.027). *Conclusions*: The use of COCs for ˃6 months is not associated with FM or BMD, regardless of dietary differences. Further attention should be paid to possible associations between COCs use, dietary habits, and body composition (including BMD).

## 1. Introduction

Combined oral contraceptives (COCs) are the most widely used contraceptive method among young women in the Western world, with more than 100 million women of reproductive age using them every day worldwide [1]. COCs are increasingly being used due to the earlier onset of sexual activity at a younger age, as well as for reasons such as acne, hirsutism, menstrual disorders, and irregular menstruation [2].

According to the World Health Organization, around 2.8 million people die every year from obesity-related causes. In 2022, there were an estimated 2.5 billion overweight adults, of whom 890 million were obese (body mass index (BMI) > 30 kg/m^2^), including 447.5 million women [3]. At the same time, as many as 18.9% of all adult women in Estonia were obese [4]. Overweight and obesity are caused by imbalances between energy intake (diet) and energy expenditure (physical activity) and result from a complex interplay of environmental, psychosocial, and genetic factors [3].

In recent years, long-term (more than 6 months) use of COCs has also been found to increase the risk of overweight and obesity in cross-sectional studies [5,6]. Klump and Di Dio (2023) suggest in their narrative review that COC-users may experience more frequent binge eating episodes (i.e., consuming a large amount of food in a short period of time, accompanied by a loss of control, overeating) compared to COC non-users [7]. However, adolescent girls (12–15 years) are also the most prone to eating disorders [8]. Weight gain has also been associated with deficiencies in essential micronutrients (calcium, phosphorus, potassium, vitamin A, vitamin B_1_, vitamin B_2_, niacin, vitamin C, and folic acid), suggesting a greater need for certain micronutrients among COC-users [6].

During adolescence, the use of COCs may also affect an important component of body composition—bone mineral density (BMD). According to Caldeirão et al. (2022), adolescents (18 ± 1.13 years) using ≤35 µg of ethinylestradiol (EE) COCs had lower BMD and bone mineral content gain after two years compared to COC non-users [9]. The results of a non-randomized clinical trial by Orsolini et al. (2023) also confirm that even healthy adolescents using COCs containing 30 μg EE are at risk of bone mass loss [10]. A meta-analysis by Goshtasebi et al. (2019) noted that although COCs now contain much lower doses of estrogen than previous years, their doses are still high enough to reduce bone turnover and, thus, are likely to inhibit bone formation, which is necessary for optimal bone growth in adolescents until maximum BMD is reached [11].

Fat mass is clearly related to both diet and physical activity [12], and this, in turn, influences BMD [13], while, as mentioned previously, different body composition components might be related to COCs intake. The interplay of diet, physical activity, COCs use, fat mass (FM), and bone health represents a complex and poorly understood association. Specifically, there is a lack of information on the dietary intake of long-term COC-users in relation to their body composition.

The ideal contraceptive should be reversible, highly effective, have minimal side effects, and be easy to administer. Although great progress has been made in recent decades in the effective targeting and optimization of physiological, pharmacological, and administrative processes for preventing pregnancy, these efforts have focused primarily on female contraceptives, leaving the development of male contraceptives on the back burner [14]. Safe contraceptives are necessary for women of all body weights, but it is known that overweight women use contraceptives less than women with a BMI below 25 kg/m^2^. Overweight women also have a higher risk of pregnancy-related hypertension, preeclampsia, gestational diabetes, cesarean section, postpartum hemorrhage [15], and thromboembolic events [16]. Therefore, it is important to continue research on the association between COCs and diet and body composition, given the global increase in overweight and obesity rates. The aim of the pilot study was to determine the food consumption of young women using COCs for >6 months and its relationship to body composition compared to non-users, and to analyze the methodology used for planning the main study.

## 2. Materials and Methods

### 2.1. Participants and Ethics Approval

The pilot study was funded by Tartu Applied Health Sciences University (TAHSU) and approved by the Research Ethics Committee of the University of Tartu (Estonia; protocol no. 382/T-2, 18 September 2023). The survey, conducted using a convenience sample from November 2023 to March 2024, was distributed via TAHSU’s social media channels, along with the enrolment slip for the Picktime system used by TAHSU. The study exclusion criteria were previous pregnancy, tobacco use, and the presence of chronic diseases that themselves, or the drugs used, could have affected body composition (including BMD). Participation in the study was voluntary for all, and participants were informed of all procedures and their rights. An informed consent form was signed bilaterally. All study participants received personalized feedback from the principal investigator regarding their individual body composition parameters (including BMD).

The study participants were young women aged 20–25 who used medium-dose COCs containing mainly 30–35 μg EE (+150 μg levonorgestrel or +1 mg desogestrel) for at least 6 months; used low-dose COCs containing ≤20 μg EE (+150 μg levonorgestrel or +3 mg drospirenone) for at least 6 months; and never used any hormonal contraceptive method. High-dose, i.e., ≥50 μg EE COCs, are not widely available in Estonia and, therefore, not used by the participants.

### 2.2. Dual-Energy X-Ray Absorptiometry

Dual-energy X-ray Absorptiometry (DXA) offers highly detailed evaluations of body composition and is recognized as the gold standard in body composition analysis. It allows for precise measurement of FM, body fat percentage (BF%), fat-free mass (FFM), and BMD across the entire body and specific regional compartments [17]. In this study, comprehensive assessments of body composition (including FM, BF%, FFM) and BMD of the whole body (WB) were determined at the TAHSU laboratory using DXA (DXA, Hologic ASY-07152; Tornik Engineering; Tijuana, Mexico). The DXA provides a Z-score, a comparison with BMD for people of the same age and sex. Initial measurements of a participant’s body height and body mass were conducted using the digital SECA 285 body scale (Hamburg, Germany); BMI was calculated as body mass (kg) divided by the body height squared (m^2^). The DXA procedures were carried out by members of the research team, who are approved by the Environmental Board of the Republic of Estonia and have received the relevant training. The study participant, in light clothing, was asked to lie on their back, and the DXA scanned their entire body. Coefficients of variation for BMD were less than 2%.

### 2.3. Questionnaires

Participants completed questionnaires to collect information on demographics, disease prevalence, medication use (including COCs), menstrual history, and lifestyle (physical activity and diet, including alcohol consumption). For the subjective assessment of physical activity, a short version of the International Physical Activity Questionnaire (IPAQ-SF), adapted into Estonian, was used. The questionnaire collects information on the frequency and duration of walking and moderate and vigorous physical activities over the past seven days. Each activity type is assigned a specific Metabolic Equivalent of Task (MET) value, which reflects its intensity relative to resting energy expenditure. The reported durations are multiplied by the corresponding MET values to calculate a total weekly MET-minutes score. Based on this score, participants were categorized into low, moderate, or high physical activity levels according to IPAQ guidelines [18].

Energy and nutrient intakes were estimated as the average of three twenty-four-hour dietary records (including two weekdays and one weekend day). Participants were instructed to maintain their usual dietary habits and daily physical activities. The Nutridata System for Research (the Health Development Institute, Estonia) [19] was used to analyze the data collected via dietary records. The nutritional analysis covered energy, carbohydrates (including fibers), proteins, fats, micronutrients, including minerals (sodium, potassium, calcium, magnesium, phosphorus, iron, zinc, iodine), and vitamins (A, B, C, D, E, K). Alcohol consumption was assessed by means of a self-assessment questionnaire where the respondent had to calculate the amount of alcohol consumed per week (1 alcohol unit = 10 g ethanol) using examples.

### 2.4. Blood Analysis

For the laboratory studies, 13.5 mL of blood (2 × 3.5 mL without additives + 2 mL with glycolysis inhibitor in the test tube and 4.5 mL EDTA (anticoagulant) blood were collected by a registered nurse. The blood analysis was performed in the TAHSU’s laboratory by bioanalysts. Minerals associated with BMD (calcium, magnesium) and vitamin D were determined. Analyses were performed on a fully automated clinical chemistry analyzer, Cobas e411 (Roche Diagnostics International AG, Rotkreuz, Switzerland) for the determination of serum vitamin D, and Cobas c111 (Roche Diagnostics International AG, Rotkreuz, Switzerland) for serum magnesium and calcium concentrations.

### 2.5. Statistical Analysis

For data processing, the software programs Sigma Plot for Windows version 11.0 (Systat Software Inc., San Jose, CA, USA) and R 2.6.2 (A Language and Environment, http://www.r-project.org, 3 November 2025) were used. To describe socio-demographic and other characteristics, and to compare COC vs. non-COC-users and medium-dose vs. low-dose EE COC-users, chi-squared for Fisher’s exact test (categorical variables), and Student’s *t*-test of the Mann–Whitney U test (continuous variables) were used. To calculate the correlation between BF% and dietary micronutrients, the Spearman test was used. To identify the effects of independent variables (use of COCs, duration of pill use, MET-min/week, BF%, the amount of calcium and fat in food) on BMD, the univariate and multiple linear regression model was used. Since only BF% was statistically significant in univariate analysis, in multiple analysis, a model was used that combined all previously used parameters to explain whether BF% remained significant against the background of other parameters. A *p*-value of <0.05 was considered significant in all analyses.

## 3. Results

### 3.1. Participants’ Characteristics and Body Composition

This pilot study presents mainly a descriptive analysis of body composition parameters and dietary intake in a cohort of 45 young women (21.8 ± 1.7 years). The cohort included both users (*n* = 26) and non-users (*n* = 19) of COCs. Within the COCs-using group, observations were made regarding the age of initiation and duration of use relative to the EE dose. The most common reasons for taking COCs were pregnancy avoidance (69.2%), management of irregular menstruation (38.5%), acne treatment (26.9%), and alleviation of painful menstruation (19.2%). Study participants were unmarried; however, approximately half (42.2%) reported being in a committed partnership at the time of data collection.

Participants’ age, anthropometric and body composition parameters, the usage of COCs, total weekly MET-minute score, and serum vitamin D and mineral (magnesium, calcium) levels are shown in Table 1. Body composition parameters (including WB BMD) and physical activity levels, as assessed by the IPAQ-SF questionnaire, did not demonstrate significant differences between groups.

### 3.2. Dietary Intake and Blood Serum Markers

Analysis of micronutrient status revealed a significant difference in serum vitamin D levels, with COC-users exhibiting higher concentrations (36.2 ± 15.3 mmol/L) compared to non-users (21.8 ± 8.6 mmol/L; *p* < 0.01; Table 1).

Alcohol consumption did not differ between study groups, and no one consumed ≥ 6 units of alcohol per week. Regarding dietary intake (Table 2), carbohydrates were the main source of energy for all participants, but the percentage of energy from fats was significantly higher for COC-users compared to non-users (*p* = 0.02). A higher proportion of COC-users received more than 80% of the recommended daily intake of vitamin B_12_ compared to non-users (*p* = 0.01). In contrast, significantly fewer COC-users had adequate vitamin C intake (*p* = 0.002).

### 3.3. Associations Between Dietary Micronutrients and Body Composition

Table 3 shows the associations of dietary micronutrient intakes with BMI and body BF%, highlighting the associations for BMI in the overall population and separately for BF% among COC-users and non-users. Significant values for BMI were found for the whole sample for iodine and calcium (coefficient −0.393, *p* = 0.008; coefficient −0.329, *p* = 0.028, respectively). The higher the BMI value, the lower the intake of iodine and calcium. The higher the BF%, the less vitamin C was obtained.

To identify independent variables associated with BMD (dependent variable), the multiple linear regression model was used for the following parameters: use of COCs, duration of COCs use, MET-min/week value, BF%, and the amount of calcium and fat in food. The result showed that BF% had a negative association with BMD (coefficient −0.008, *p* = 0.027).

## 4. Discussion

The results of the pilot study show that the use of COCs for ˃6 months is not associated with FM or BMD, regardless of dietary differences. COC-users obtain a significantly higher percentage of their energy from fats compared to non-users. This observation warrants further investigation in the context of hormonal influences on dietary choices. The existing literature suggests that increased intakes of fat [20], carbohydrate [21], or both [22] have been observed during the pre-menstrual period, a phase characterized by elevated progesterone levels. In COC-users, progesterone levels remain consistently elevated throughout the COC-use period (21–24 days), potentially extending this hormonal influence on dietary preferences [23]. Unfortunately, the exact phase of the menstrual cycle of the subjects in our pilot study was not investigated. In addition, the subjects in our study used COC preparations with different progestins. Since lower body mass has been found in users of COCs containing drospirenone than in users of preparations containing desogestrel [24], it is important to consider the exact composition of COC preparations when designing the study and recruiting subjects.

Despite references to an increase in FM and even the development of overweight and obesity among COC-users [5,6,25,26], based on the results of our pilot study, there were no differences between COC-users and non-users in terms of body composition components (including BMD). It should be considered that the teenage years, when our participants started taking COCs, are a crucial time to reach peak BMD [27], as around 90–95% of bone mass is typically reached by the age of 18 in young women [28]. It has been found that adolescents taking COCs (especially 30–35 µg EE) accumulate less bone mass than healthy controls [29,30,31]. Bone demineralization starts to occur faster than bone mineralization as early as 25 years of age [32]. Therefore, the use of a contraceptive method that can reduce bone mineralization in women under 25 years of age can have potentially negative long-term effects on bone health [33].

Although deficiencies of certain micronutrients (phosphorus, potassium, vitamin A, vitamin B_1_, vitamin B_2_, and vitamin C) may be associated with obesity in COC-users [6], in our study, most of these micronutrients were obtained at the same levels as non-users and were not associated with the BMI and BF%. The exception is vitamin C, which a large proportion of COC-users did not receive in adequate amounts, while there were no associations between the vitamin and BMI and BF%.

BMD is closely related to vitamin D availability [34]. The best indicator of vitamin D status in humans is the blood level of 25-hydroxycholecalciferol (25(OH)D), as it reflects both skin synthesis and dietary intake [35]. The results of our study, which demonstrated significantly higher serum vitamin D levels in COC-users compared to non-users, align with findings reported by Öberg et al. (2022) in a study of adolescent girls [36], reinforcing the potential protective effect of COCs against vitamin D deficiency [37]. The Norwegian research also revealed significantly elevated 25(OH)D levels among COC-users, leading the authors to propose that the estrogen component of COCs stimulates hepatic production of vitamin D-binding protein and influences vitamin D metabolism. This is explained by the direct effect of estrogen on the activity of enzymes involved in vitamin D metabolism. Estrogen may increase the conversion of vitamin D to its active form, which may contribute to higher serum vitamin D levels in COC-users [38]. It has also been suggested that hormonal contraceptives may increase vitamin D absorption in the gut and lead to higher circulating levels of vitamin D [39].

The pilot study has several limitations, mainly the cross-sectional study design and small sample size (including the unequal number of subjects in the study groups), which restricts the ability to draw broad conclusions and may limit the generalizability of the findings. A significant limitation is the use of COCs containing different progestins, which could differentially influence appetite and body composition. It would be valuable to further investigate the associations between COCs intake, dietary habits, body composition, and hormones such as leptin and ghrelin, which are involved in appetite regulation. For example, in women with polycystic ovary syndrome, oral contraceptives containing EE and drospirenone have been found to increase levels of ghrelin but not leptin after 3 months of use [40]. However, ghrelin stimulates appetite and the imagination of food [41], while leptin is one of the main appetite suppressants [42].

Despite these limitations, a key strength of this pilot study is its focus on a homogeneous group of participants—Caucasian women within a narrow age range who had never smoked, been pregnant, or had any medical conditions affecting body composition (including BMD). This controlled group helps to ensure that observed effects are less confounded by external factors. Henceforth, it would be beneficial to specify the types and formulations of COCs used, and to examine the long-term effects—over periods of six months, one year, and three years—of different EE concentrations and progestin types on dietary intake and body composition (including BMD). Including assessments across menstrual cycle phases and measuring adipokines like leptin and ghrelin could provide deeper insights into hormonal effects on appetite and body composition. When planning a study, it is essential to perform a preliminary power calculation to ensure that the number of subjects studied is sufficient to allow generalizations to be made and clinical recommendations to be given later.

## 5. Conclusions

The use of COCs for ˃6 months is not associated with FM or BMD, regardless of dietary differences. Since BF% is negatively associated with BMD, attention should be paid to possible associations between COCs use, dietary habits, body composition, and BMD.

## Figures and Tables

**Table 1:** Participants’ age, anthropometric, and body composition parameters, the usage of combined oral contraceptives (COCs), and total weekly MET-minute score.

Parameters	All Participants *n* = 45	COC Non-User *n* = 19	COC-User *n* = 26	15–20 EE *n* = 18	30–35 EE *n* = 8
Age, years (mean; SD)	21.8; 1.7	21.6; 1.5	21.9; 1.8	22.1; 2.0	21.5; 1.4
Age at onset of COC use, years (mean; SD)			17.2; 1.8	16.9; 1.9	17.8; 1.5
Duration of COC use, years (mean; SD)			4.3; 2.0	4.8; 2.1	3.3; 1.2
Body height, cm (mean; SD)	168.3; 5.6	168.8; 6.5	167.9; 5.0	167.8; 5.3	167.9; 4.5
Body mass, kg (mean; SD)	62.5; 8.5	62.1; 1.0	62.4; 7.0	63.4; 7.8	60.2; 4.3
Body mass index, kg/m^2^ (mean; SD)	22.1; 3.1	21.9; 3.1	22.3; 3.1	22.5; 3.2	21.4; 3.1
Body composition					
Fat mass, kg (mean; SD)	21.7; 7.5	21.3; 5.2	22.0; 4.9	22.9; 5.5	19.8; 2.2
BF% (mean; SD)	34.5; 4.6	33.9; 3.9	35.0; 5.2	35.9; 5.5	32.9; 3.7
Fat-free mass, kg (mean; SD)	38.6; 5.1	39.0; 6.0	38.3; 4.3	38.3; 4.6	38.2; 3.9
WB BMD, g/cm^2^ (mean; SD)	1.12; 0.11	1.12; 0.12	1.11; 0.11	1.10; 0.08	1.15; 0.15
WB Z-score (mean; SD)	0.15; 1.39	0.12; 1.5	0.18; 1.33	−0.22; 1.04	0.63; 1.83
Physical activity level based on MET-min/week					
MET-min/week (mean; SD)	3838.1; 2655.6	3567.8; 2435.3	4035.7; 2836.5	4484.9; 3183.4	3024.9; 1566.3
Low (%)	15.6	21.1	11.5	5.6	25.0
Moderate (%)	26.7	21.1	30.8	33.3	25.0
High (%)	57.8	57.9	57.7	61.1	50.0
Micronutrients in blood					
Calcium, mmol/L (mean; SD)	2.3; 0.2	2.3; 0.3	2.3; 0.3	2.3; 0.3	2.4; 0.1
Magnesium, mmol/L (mean; SD)	0.8; 0.2	0.8; 0.1	0.8; 0.1	0.8; 0.1	0.8; 0.1
Vitamin D, nmol/L (mean; SD)	30.1; 14.6	21.8; 8.6	36.2; 15.3 *	39.4; 16.5	29.0; 9.0

COC—combined oral contraceptive; EE—ethinylestradiol; WB BMD—whole body bone mineral density; BF%—body fat percentage; MET—Metabolic Equivalent Task; Vitamin D—serum 25-hydroxyvitamin D; *—*p* ≤ 0.05.

**Table 2:** Average daily energy intake, macronutrient distribution, and micronutrients in diet.

Parameters	All Participants *n* = 45	COC Non-User *n* = 19	COC-User *n* = 26	15–20 EE *n* = 18	30–35 EE *n* = 8
Energy, kcal (mean; SD)	1558.5; 515.5	1565.1; 492.5	1553.7; 541.3	1535.9; 589.0	1593.8; 448.7
Carbohydrates, g (mean; SD)	179.6; 66.8	189.2; 64.9	172.6; 71.9	161.8; 68.8	196.9; 77.6
% of total energy	43.7; 8.9	46.1; 9.7	42.0; 8.0	40.1; 6.9	46.2; 9.1
Fibers, g (mean; SD)	16.8; 9.0	19.3; 10.4	15.1; 7.4	14.2; 7.0	17.1; 8.4
Fat, g (mean; SD)	66.1; 25.7	60.4; 23.2	70.2; 27.1	72.0; 30.6	66.2; 17.9
% of total energy	38.2; 7.0	34.4; 6.6	40.9; 6.1 *	42.0; 4.3	38.5; 8.8
Protein, g (mean; SD)	69.4; 35.6	76.2; 44.4	64.4; 27.3	65.4; 29.3	62.1; 23.9
% of total energy	17.4; 5.2	18.6; 6.7	16.6; 3.6	17.1; 4.0	15.3; 2.3
Micronutrients in diet					
Calcium, mg (mean; SD)	668.8; 404.2	771.6; 524.4	593.7; 275.1	626.1; 317.4	520.7; 129.7
<80% of the recommended (%)	71.1	63.2	76.9	66.7	100
Iodine, <80% of the recommended (%)	68.9	68.4	69.2 *	55.6	100
Iron, <80% of the recommended (%)	88.9	89.5	88.5	88.9	87.5
Magnesium, mg (mean; SD)	232.6; 105.2	256.9; 124.6	214.8; 86.8	213.3; 91.6	218.3; 80.8
<80% of the recommended (%)	60.0	52.6	65.4	66.7	62.5
Potassium, <80% of the recommended (%)	73.3	68.4	76.9	77.8	75
Phosphorus, <80% of the recommended (%)	4.4	5.3	3.8	5.6	0.0
Zinc, <80% of the recommended (%)	46.7	42.1	50.0	50.0	50.0
Sodium, <80% of the recommended (%)	0.0	0.0	0.0	0.0	0.0
Vitamin A, <80% of the recommended (%)	73.3	73.7	73.1	61.1	100
Vitamin B_12_, µg (mean; SD)	3.7; 2.5	3.5; 2.2	3.9; 2.8 *	3.5; 2.1	4.8; 3.9
<80% of the recommended (%)	55.6	68.4	46.2	50.0	37.5
Vitamin C, <80% of the recommended (%)	66.7	52.6	76.9 *	88.9	50.0
Vitamin D, μg (mean; SD)	4.2; 4.4	4.0; 4.8	4.3; 4.1	4.8; 4.7	3.3; 2.5
<80% of the recommended (%)	84.4	84.2	84.6	77.8	100

COCs—combined oral contraceptives; EE—ethinylestradiol; *—*p* ≤ 0.05.

**Table 3:** Dietary micronutrient correlations with BMI and body fat percentage (BF%).

	BMI All Participants *n* = 45		BF% All Participants *n* = 45		BF% COC-User *n* = 26		BF% COC Non-User *n* = 19	
	r=	*p*=	r=	*p*=	r=	*p*=	r=	*p*=
Calcium	**−0.329**	**0.028**	−0.130	0.391	−0.264	0.191	0.123	0.610
Iodine	**−0.393**	**0.008**	−0.114	0.456	−0.182	0.371	−0.119	0.620
Iron	−0.194	0.199	−0.123	0.418	−0.164	0.418	−0.068	0.776
Magnesium	−0.253	0.093	−0.245	0.104	−0.262	0.194	−0.007	0.974
Sodium	−0.177	0.244	0.092	0.548	0.255	0.207	0.095	0.694
Phosphorus	−0.221	0.144	−0.160	0.292	−0.246	0.223	0.137	0.570
Potassium	−0.161	0.290	−0.181	0.232	−0.203	0.315	0.086	0.721
Zinc	−0.085	0.577	−0.032	0.834	−0.037	0.854	0.086	0.721
Vitamin A	−0.120	0.429	−0.072	0.639	−0.084	0.678	−0.051	0.832
Vitamin B_1_	−0.035	0.818	0.060	0.697	−0.141	0.490	0.267	0.264
Vitamin B_2_	−0.026	0.867	<0.001	0.998	−0.027	0.894	0.047	0.843
Vitamin B_6_	−0.034	0.826	−0.062	0.683	−0.147	0.469	0.204	0.397
Vitamin B_9_	−0.161	0.280	−0.162	0.285	−0.0273	0.175	0.168	0.484
Vitamin B_12_	0.021	0.891	0.114	0.454	−0.069	0.736	0.319	0.179
Vitamin C	−0.044	0.775	**−0.326**	**0.029**	−0.275	0.710	−0.153	0.526
Vitamin D	−0.039	0.809	0.134	0.379	−0.120	0.555	0.282	0.236
Vitamin E	−0.223	0.140	0.104	0.494	0.046	0.823	0.309	0.194
Vitamin K	−0.100	0.522	0.134	0.380	−0.450	0.825	0.218	0.365

BMI—body mass index; BF%—body fat percentage; COC—combined oral contraceptive; **in bold**, statistically significant correlations.

## Data Availability

Data is unavailable due to privacy and ethical restrictions.

## Abbreviations

The following abbreviations are used in this manuscript:

COC	combined oral contraceptive
EE	ethinylestradiol
DXA	dual-energy X-ray absorptiometry
BMD	bone mineral density
WB	whole body
BMI	body mass index
FM	fat mass
FFM	fat-free mass
BF%	body fat percentage
TAHSU	Tartu Applied Health Sciences University
IPAQ-SF	Physical Activity Questionnaire
MET	metabolic equivalent of task
